# *CHITINASE LIKE1* Regulates Root Development of Dark-Grown Seedlings by Modulating Ethylene Biosynthesis in *Arabidopsis thaliana*

**DOI:** 10.3389/fpls.2019.00600

**Published:** 2019-05-14

**Authors:** Shin-Yuan Gu, Long-Chi Wang, Chiao-Mei Cheuh, Wan-Sheng Lo

**Affiliations:** ^1^Institute of Plant and Microbial Biology, Academia Sinica, Taipei, Taiwan; ^2^Department of Life Sciences, National Central University, Taoyuan City, Taiwan; ^3^Department of Life Sciences, National Chung Hsing University, Taichung, Taiwan

**Keywords:** ACC synthase, acsinones, CTL1, chemical genetics, ETO1, ACC oxidase

## Abstract

The plant hormone ethylene plays a regulatory role in development in light- and dark-grown seedlings. We previously isolated a group of small-molecule compounds with a quinazolinone backbone, which were named acsinones (for ACC synthase inhibitor quinazolinones), that act as uncompetitive inhibitors of 1-aminocyclopropane-1-carboxylic acid (ACC) synthase (ACS). Thus, the triple response phenotype, which consists of shortened hypocotyls and roots, radial swelling of hypocotyls and exaggerated curvature of apical hooks, was suppressed by acsinones in dark-grown (etiolated) *ethylene overproducer* (*eto*) seedlings. Here, we describe our isolation and characterization of an Arabidopsis *revert to eto1 9* (*ret9*) mutant, which showed reduced sensitivity to acsinones in etiolated *eto1* seedlings. Map-based cloning of *RET9* revealed an amino acid substitution in *CHITINASE LIKE1* (*CTL1*), which is required for cell wall biogenesis and stress resistance in Arabidopsis. Etiolated seedlings of *ctl1^ret*9*^* showed short hypocotyls and roots, which were augmented in combination with *eto1-4*. Consistently, *ctl1^ret*9*^* seedlings showed enhanced sensitivity to exogenous ACC to suppress primary root elongation as compared with the wild type. After introducing *ctl1^ret*9*^* to mutants completely insensitive to ethylene, genetic analysis indicated that an intact ethylene response pathway is essential for the alterations in root and apical hook but not hypocotyl in etiolated *ctl1^ret*9*^* seedlings. Furthermore, a mild yet significantly increased ethylene level in *ctl1* mutants was related to elevated mRNA level and activity of ACC oxidase (ACO). Moreover, genes associated with ethylene biosynthesis (*ACO1* and *ACO2*) and response (*ERF1* and *EDF1*) were upregulated in etiolated *ctl1^ret*9*^* seedlings. By characterizing a new recessive allele of *CTL1*, we reveal that CTL1 negatively regulates ACO activity and the ethylene response, which thus contributes to understanding a role for ethylene in root elongation in response to perturbed cell wall integrity.

## Introduction

Chitinases (EC 3.2.1.14) are a group of enzymes that catalyze the hydrolysis of chitin by cleaving the β-1,4 linkage of *N*-acetylglucosamine. Plant chitinases and chitinase-like (CTL) proteins have diverse functions mostly in cell wall biosynthesis and disease resistance (Collinge et al., 1993). The Arabidopsis (*Arabidopsis thaliana*) genome contains two paralogous sequences encoding *CTL1* and *CTL2*, which were classified into class II of family 19 chitinases. Due to lack of conserved amino acid residues required for chitin binding and catalytic activity, both CTL proteins do not have a chitinase activity (Hermans et al., 2010; Hossain et al., 2010). Mutations in different alleles of *CTL1* result in several developmental defects, including semi-dwarfism, ectopic deposition of lignin in pith (*elp1*), reduced elongation of roots and hypocotyls, abnormal cell expansion in roots (*pom1*), ectopic root hairs (*erh2*), ethylene overproduction and aberrant cell shape with incomplete cell walls (*ctl1*) (Hauser et al., 1995; Schneider et al., 1997; Zhong et al., 2002; Sanchez-Rodriguez et al., 2012). Analysis of the *anion-related root morphology* (*arm*) mutant, an allele of *ctl1*, showed that CTL1 modulates the plastic development of the root system architecture under high nitrate, sucrose, and chloride conditions. Thus, the *arm* mutant has reduced primary root length, radial swelling of roots and increased number of lateral roots and root hairs (Hermans et al., 2010, 2011). In addition, characterization of the Arabidopsis *hot2* mutant, an allele of *ctl1*, revealed *CTL1* involved in tolerance to heat, salinity and drought stresses (Hong et al., 2003; Kwon et al., 2007).

*CTL2* is a paralog of *CTL1* in Arabidopsis and shares 70% amino acid similarity (Hossain et al., 2010). Despite the distinct spatial and temporal expression patterns of *CTL1* and *CTL2*, *CTL2* completely complements *ctl1* under control of the *CTL1* promoter, which suggests that *CTL1* and *CTL2* are functionally equivalent (Hossain et al., 2010; Sanchez-Rodriguez et al., 2012). CTL1 is secreted to the apoplast and co-localizes with cell wall cellulose synthases (CESAs) in the endomembrane system (Sanchez-Rodriguez et al., 2012). Transcriptome data revealed that *CTL1* and *CTL2* are co-expressed with primary and secondary CESAs, respectively, in different plant species (Persson et al., 2005; Wu et al., 2012). Mutations in *CTL1* reduce the movement of CESAs and cellulose content. Both CTL1 and CTL2 bind glucan polymers and act as a scaffold to establish interactions between cellulose microfibrils and hemicelluloses. The *ctl1ctl2* double mutant shows reduced crystalline cellulose content in the cell wall, so CTL1 and CTL2 are important for cellulose production and determining cell wall rigidity in Arabidopsis (Sanchez-Rodriguez et al., 2012).

When plants encounter nutrient deficiency, the morphologic or physiologic alterations of the roots facilitate the mobilization and uptake of nutrients. Plants exhibit plasticity in root development responding to nutrient deficiency by altering the length, number and angle of roots and root hairs for nutrient acquisition (Shahzad and Amtmann, 2017). The plant hormone ethylene participates in both root morphology and the physiological response under inadequate nutrients (Garcia et al., 2015). Ethylene negatively regulates root elongation, lateral root development and gravitropic responses but positively controls the frequency of root waving and stimulates root hair formation (Buer et al., 2006; Swarup et al., 2007; Negi et al., 2008). In Arabidopsis, ethylene is involved in lateral root development by regulating nitrate transporters under the excess nitrate condition (Khan et al., 2015). When plants are exposed to environmental transition from high to low nitrate, a rapid burst of ethylene is detected, accompanied by reduced length and number of lateral roots (Tian et al., 2009). In addition, ethylene mediates altered root development under limited phosphorus by inhibiting primary root elongation but promoting lateral roots and enhancing root hair outgrowth to improve phosphorus acquisition (Neumann, 2015). However, ethylene level is increased under excess iron to antagonize the iron-induced inhibition of primary root growth arrest (Li G. et al., 2015). Ethylene may actively participate in modifying root architecture in response to environmental changes.

Ethylene is a simple hydrocarbon gas that regulates a number of physiological and developmental events in plants (Wang et al., 2002). Ethylene gas is derived from methionine by a three-step process that requires *S*-adenosyl methionine synthase (SAMS), 1-aminocyclopropane-1-carboxylic acid synthase (ACC synthase; ACS) and ACO. The conversion of SAM to ACC vis ACS is considered the rate-limiting step in ethylene biosynthesis (Kende, 1989). ETHYLENE OVERPRODUCER1 (ETO1) is a negative regulator in ethylene biosynthesis by inhibiting the enzymatic activity and protein stability of ACS5 via 26S proteasome-mediated degradation (Wang et al., 2004). When exposed to excess ethylene, dark-grown (etiolated) Arabidopsis seedlings display a triple response phenotype of shortened hypocotyls and roots, radial swelling of hypocotyls and exaggerated curvature of apical hooks (Guzman and Ecker, 1990). Many, if not all of the key regulatory components in the ethylene biosynthesis and signaling pathways have been identified mainly by characterizing Arabidopsis mutants with the mis-regulated triple response phenotype (Guzman and Ecker, 1990; Chang et al., 1993; Roman et al., 1995). The ethylene signaling cascade is initiated by ethylene binding to a group of endoplasmic reticulum (ER) membrane-bound receptors including ETHYLENE RESPONSE1 (ETR1), ETR2, ETHYLENE RESPONSE SENSOR1 (ERS1), ERS2, and ETHYLENE INSENSITIVE4 (EIN4), all structurally related to bacterial two-component histidine kinase sensors (Bleecker et al., 1998; Chen et al., 2002). In the absence of ethylene, these receptors act as negative regulators of the ethylene response by activating CONSTITUTIVE TRIPLE RESPONSE1 (CTR1), a serine/threonine Raf-like kinase, to suppress the ethylene response via phosphorylation of ER membrane-bound EIN2 (Kieber et al., 1993; Alonso et al., 1999). When ethylene binds to receptors, CTR1 is inactivated and fails to phosphorylate EIN2, which results in proteolysis of EIN2 to release a protein fragment consisting of the cytosolic carboxyl terminus of EIN2 (EIN2C) to evoke the ethylene response (Ju et al., 2012; Qiao et al., 2012; Li W. et al., 2015). The transcription factors EIN3 and EIN3-LIKE1 (EIL1) are the key nuclear regulators that initiate a transcriptional cascade of ethylene response in Arabidopsis (Chao et al., 1997; Chang et al., 2013). EIN3 and EIL1 are degraded by the 26S proteasome system dependent on EIN3 binding F-Box proteins, EBF1 and EBF2 (Guo and Ecker, 2003; Potuschak et al., 2003; Gagne et al., 2004). Nuclear translocation of EIN2C promotes the protein stability of EIN3 and EIL1 by downregulating EBF1 and EBF2 (Li W. et al., 2015; Merchante et al., 2015). Recently, EIN2C was shown to modify histone H3 acetylation to facilitate gene expression involved in ethylene response (Zhang et al., 2016, 2017).

The chemical genetics methodology starts with chemical screens of small-molecule compounds, followed by genetic studies and has been used as an alternative strategy to conventional genetic screening for discovery of new components involved in many aspects of plant physiology (Dejonghe and Russinova, 2017). Use of small-molecule compounds for functional studies offers several advantages over conventional genetic methods, such as reversible, instantaneous and conditional alterations for phenotypes of interest. Furthermore, use of small molecules provides a solution to genetic mutants involving gene redundancy, genetic lethality and pleiotropism (Blackwell and Zhao, 2003; Toth and van der Hoorn, 2010). Studying genetic mutants with altered sensitivity to the chemical compounds of interest provides a means to discover novel regulatory components in plant hormone signal transduction pathways. The long-sought abscisic acid receptor PYRABACTIN RESISTANCE1 (PYR1) was revealed after identification of a synthetic compound, pyrabactin, by a chemical genetic screen (Park et al., 2009). Recently, chemical genetics has been used to discover new analogs, agonists, and inhibitors that disrupt biosynthesis or signaling networks of plant hormones, such as those involved in auxin, abscisic acid, brassinosteroid, strigolactone, and ethylene functions (De Rybel et al., 2009; Park et al., 2009; He et al., 2011; Holbrook-Smith and McCourt, 2018).

Previously, we identified 74 small molecules that affect the ethylene phenotype in *eto1-4* to differential degrees by screening 10,000 structurally diverse chemical compounds. We selected three hit compounds sharing a common quinazolinone backbone that effectively reduced ethylene level and suppressed the triple response phenotype in etiolated *eto1-4* for further characterization. These compounds are novel uncompetitive inhibitors of ACS and were named acsinones (for ACS inhibitor quinazolinones) (Lin et al., 2010). Subsequently, we uncovered 19 independent Arabidopsis mutants showing reduced sensitivity to acsinone7303 in etiolated *eto1* seedlings, which were called *revert to eto1*, *ret*. We reported that *ret8* and *ret41* are new alleles of *CELLULOSE SYNTHASE6* (*CESA6*) and *DE-ETIOLATION2* (*DET2*), respectively (Chen et al., 2013). Mutations in *CESA6/RET8* and *DET2/RET41* show defects in cell wall rigidity and brassinosteroid biosynthesis, respectively (Chory et al., 1991; Fagard et al., 2000; Chen et al., 2013).

Here we report the molecular cloning and functional characterization of *ret9*. By using map-based cloning combined with a whole-genome sequencing approach, we revealed that *ret9* bears a mutation in the *CTL1* locus that encodes a chitinase-like protein. Further studies demonstrated mutations in *CTL1* resulting in elevated ethylene level and enhanced responsiveness to ethylene in reducing root elongation in etiolated seedlings. We present results to support a role for *CTL1* in regulating ethylene biosynthesis and sensitivity during root development.

## Materials and Methods

### Plant Material and Growth Conditions

All plants were derived from the wild-type Arabidopsis (*A. thaliana*) Columbia ecotype (*Col-0*) and cultivated in growth chambers at 22°C and under 100–150 μE m^-2^s^-1^ illumination with 16-h light/8-h dark conditions. The ethylene mutants *etr1-1* (Chang et al., 1993), *ein2-47* (SALK_086500C), *ein3-1eil1-1* (Alonso et al., 2003) were obtained from the Arabidopsis Biological Resource Center (Columbus, OH, United States). Seeds of *ctl1-1* (SALK_093049) was kindly provided by Dr. Staffan Persson (The University of Melbourne, Parkville, Australia) (Sanchez-Rodriguez et al., 2012). The *eto1-4* mutant carried a luciferase (LUC) reporter system under the control of five copies of the EIN3-binding sites (*pro35Smin::5xEBS-LUC*) and was described previously (Lin et al., 2010). Preparation of growth medium and the phenotype-based screening for *ret* mutants were performed as described (Chen et al., 2013). An amount of 5 μM aminoethoxyvinylglycine (AVG) (Sigma), 10 μM sodium thiosulfate (STS) (Sigma), 10 μM acsinone7303 (ChemBridge) and ACC (Merck) were used unless otherwise indicated. To generate mutants combined with *ctl1^ret*9*^*, the *ret9* mutant (*eto1-4ctl1^ret*9*^*) was crossed separately with *etr1-1*, *ein2-47*, and *ein3-1eil1-1* mutants. After selfing of F1 plants, the double (*ctl1^ret*9*^etr1-1*), (*ctl1^ret*9*^ein2-47*) and triple (*ctl1^ret*9*^ein3-1eil1-1*) homozygotes were identified in the F2 or F3 generation by PCR-based genotyping. Images of Arabidopsis seedlings were photographed by using a digital camera (Canon Powershot A620) attached to a stereomicroscope (Zeiss Discovery V8). ImageJ^1^ was used to measure rosette diameter, the length of hypocotyls and roots. All primers used in this report for genotyping, sequencing, cloning of genes and cDNA and RT-qPCR are in Supplementary Table S3.

### Map-Based Cloning of *ret9* Mutant and Genetic Analysis

To isolate *ctl1^ret*9*^*, the *ret9* mutant was crossed with the wild type (*Col-0*) to remove the *eto1-4* allele from the genetic background. For gene mapping, the *ctl1^ret*9*^* mutant was crossed with *Landsberg erecta* (*Ler*) to generate F1 plants to collect F2 seeds for the mapping population. Genomic DNA was extracted for PCR-based gene mapping from individual F2 plants with a *ret9* phenotype. Markers for mapping *ctl1^ret*9*^* were referred from the Monsanto Arabidopsis Polymorphism collection^2^ and Arabidopsis Mapping Platform^3^. Primers were designed to isolate candidate genes by PCR with use of Phusion DNA polymerase (Finnzymes). The PCR products were cloned into pJET1.2 (Fermentas) and analyzed by sequencing. The *ctl1^ret*9*^* allele was identified and confirmed by comparing the sequences in the *ret9* mutant in independent plants. For genetic analysis, the *ret9* mutant was backcrossed twice, and homozygous F2 or F3 plants were used for phenotypic characterization. For allelic analyses, the *ret9* mutant was crossed with *ctl1-1*, and F1 plants were examined. Plants with the desired genotype were selected from F1 or F2 progeny of crosses and verified by PCR-based genotyping with derived cleaved amplified polymorphic sequence primers after scoring the phenotype of etiolated seedlings (Neff et al., 1998).

### Cryo-Scanning Electron Microscopy and Confocal Microscopy

Three-day-old etiolated seedlings of *Col-0*, *eto1-4*, *ctl1-1*, and *ctl1^ret*9*^* were mounted on a specimen holder, then cryo-fixed in liquid nitrogen slush. Samples were observed in high vacuum mode on a cryo-stage maintained at -190°C with a cryo-scanning electron microscope (FEI Quanta 200 SEM/Quorum Cryo System PP2000TR FEI) at 20 kV. Roots of 3-day-old etiolated seedlings were stained with 10 mg/ml propidium iodide (PI) solution and analyzed by using a confocal laser scanning microscope (Zeiss LSM880 with Airyscan).

### Complementation Analysis of *ret9*

A 2199-bp genomic DNA fragment containing a 578-bp upstream sequence and a 330-bp downstream sequence of the *CTL1* locus (*gCTL1*) was amplified by PCR and cloned into pCAMBIA1300 by *BamH*I and *Sal*I sites (CAMBIA, Canberra). The DNA construct was introduced into *ret9* mutant by *Agrobacterium*-mediated transformation with the *GV3101* strain by the floral dip method (Clough and Bent, 1998). Transgenic plants were selected on Murashige and Skoog (MS) agar media (pH 5.7) supplemented with 25 μg ml^-1^ hygromycin B. The homozygosity of *ctl1^ret*9*^* was confirmed by genotyping, and the complementation of *ret9* by a genomic copy of *CTL1* was verified in the T2 generation based on co-segregation of the wild-type phenotype and hygromycin resistance.

### Live Imaging and Quantification of Luciferase Activity

Imaging of luciferase activity was performed as described (Lin et al., 2010) with minor modifications. Approximately 20 seeds were sown and germinated on MS agar media supplemented with chemicals specified in individual treatments. After 3 days of germination in the dark, etiolated seedlings were sprayed with luciferin (2 mM, Biosynth International), then kept in the dark for 5 min before imaging by using the Xenogen IVIS System (Caliper Life Sciences). Approximately 750 of 3-day-old etiolated seedlings were grounded in liquid nitrogen and extracted with Luciferase Cell Culture Lysis Reagent (Promega) to obtain cell lysates. Protein concentrations of cell lysates were quantified by Bradford assay. The luminescence was measured in 96-well microtiter plates by using the Synergy Mx 3M Microplate Reader (BioTek) immediately after mixing 10 μl cell lysates with 50 μl Luciferase Assay Reagent.

### Enzyme Activity and Ethylene Measurement

Assay of ACS and ACO activity was performed as described (Larsen and Cancel, 2004) with minor modification. For ACS activity assay, total proteins from 3-day-old etiolated seedlings were powdered with liquid nitrogen and extracted in 2 volumes (w/v) of buffer containing 100 mM HEPES (pH 8.0), 10 μM pyridoxal phosphate, 5 mM DTT, 1 mM EDTA, 10 μg ml^-1^ leupeptin, 10 μg ml^-1^ pepstatin A, and 1 mM phenyl methane sulfonyl fluoride (PMSF). The enzymatic reaction was started by adding 1.25 mM *S*-adenosyl-L-methionine with 1.5 mg total protein extract and incubated at 25°C for 30 min. The ACC formed was chemically converted to ethylene by the addition of HgCl, followed by a 1:1 mix of saturated NaOH:bleach. For ACO activity assay, total protein was extracted from 3-day-old etiolated seedlings as described above in 2 volumes (w/v) of buffer containing 100 mM Tris-HCl (pH 7.5), 10% glycerol, 30 mM ascorbate acid, 0.1 mM FeSO_4_, 20 mM NaHCO_3_, 5 μM AVG, and aforementioned protease inhibitors. The enzymatic reaction was started by adding 1 mM ACC with 1.5 mg total protein extract and incubation at 30°C for 1 h. Ethylene production from Arabidopsis seedlings was analyzed as described (Chen et al., 2013). Sterilized seeds were sown in 10-ml gas chromatograph (GC) crimp-top vials (approximately 30 seeds per vial) containing 0.5% MS medium (pH 5.7) and germinated in the dark for 3 days. Accumulated ethylene was measured from the headspace of the GV vials by using a GC instrument (HP6890, Agilent Technologies) equipped with a capillary column (CP7381, Varian) and an autosampler (HP7694, Agilent Technologies).

### Protein Extraction and Immunoblot Analysis

Arabidopsis tissue was ground to a fine powder in liquid nitrogen by using a mortar and pestle. Total protein was extracted with lysis buffer containing 0.1% Nonidet P-40, 50 mM Tris-HCl, pH 7.5, 150 mM NaCl, 10 mM MgCl_2_, 1 mM phenyl-methanesulfonyl fluoride and protease inhibitor cocktail (Roche). Proteins were separated by 12% SDS-PAGE and transferred to nitrocellulose membrane (Sartorius Biotech) by electroblotting for 1.5 h at 100 mA. Proteins were detected by immunoblotting with anti-ACO antibody (sc-12781, Santa Cruz Biotechnology) and anti-RPN10 antibody (Lin et al., 2011). Blots were developed with horseradish peroxidase-linked secondary antibodies and Western Bright ECL reagent (Advansta).

### Plant RNA Extraction and Quantitative RT-PCR

Total RNA was prepared from 3-day-old etiolated seedlings by using the Plant Total RNA Miniprep Purification Kit (GeneMark) following the manufacturer’s protocol. To generate cDNA, 2 μg total RNA was treated with RQ1 DNase (Promega) and converted to cDNA by using MMLV reverse transcriptase (TOYOBO) according to the manufacturer’s instruction. Quantitative PCR involved use of an ABI QuantStudio 12 K Flex Real Time PCR system (Applied Biosystems) with SYBR Green Master Mix (Fast Power SYBR Green; Applied Biosystems). The sequences of *ACO1*, *ACO2*, *ERF1* and *EDF1* primers used in RT-qPCR were described previously (Schellingen et al., 2014). Expression of Arabidopsis *UBQ10* was an internal reference.

### Statistical Analysis

Data are expressed as mean ± SD. Statistical analysis were performed with analysis of variance (ANOVA) test with Duncan *post hoc* test by the SPSS program (IBM Co., Armonk, NY, United States) to compare differences between more than two groups or treatments. The comparisons between two groups were conducted using two-tailed student’s *t*-test.

## Results

### *CTL1* Is Mutated in the *ret9* Mutant

We previously identified a group of small molecules termed acsinones that suppressed the constitutive triple response phenotype in etiolated *eto1* seedlings (Lin et al., 2010). To uncover additional physiological roles of acsinones and potential signaling components in the ethylene response, we used a chemical genetics approach to screen Arabidopsis mutants with altered sensitivity to acsinones. The mutants, named *ret* (*revert to eto1*), show reduced sensitivity to acsinones that restored the etiolated *eto1* seedling phenotype (Supplementary Figure S1) (Chen et al., 2013). Here, we characterized one of the *ret* mutants, *ret9*, isolated from ethyl methanesulfonate-mutagenized *eto1-4* seeds. The *ret9* mutant was backcrossed to *eto1-4* for genetic analysis. The phenotype of F_2_ progeny segregated at an approximately 3:1 ratio of *eto1-4* to *ret9* in the presence of acsinone7303, which suggests that *ret9* is a recessive mutation in a single locus (Table 1). To uncover the mutation in *ret9* by a map-based cloning approach, we generated a mapping population by crossing *ret9* (in *Col-0* background) with *Landsberg erecta* and collected F_2_ seeds from self-pollinated F_1_ plants. The F_2_ progeny with the *ret9* phenotype were selected for further genotyping and mapping by using simple sequence length polymorphism and cleaved amplified polymorphic sequence PCR-based molecular markers (available from the Monsanto Arabidopsis Polymorphism collection^4^) (Supplementary Table S3).

**Table 1:** Genetic analysis of *ret9*.

Strains and crosses (♂ × ♀)	Generation	Total	Sensitivity to acsinone7303			
			Yes^a^	No	χ^2b^	*P*
*eto1-4*/*eto1-4*		35	35	0		
*eto1-4*/*eto1-4* × *ret9*/*ret9*^c^	F1	48	48	0		
	F2	203	146	57	1.03	0.31
*ctl1-1*/*ctl1-1*^c^		40	0	40		
*ret9*/*ret9* × *ctl1-1*/*ctl1-1*	F1	43	0	43		


**FIGURE 1:**
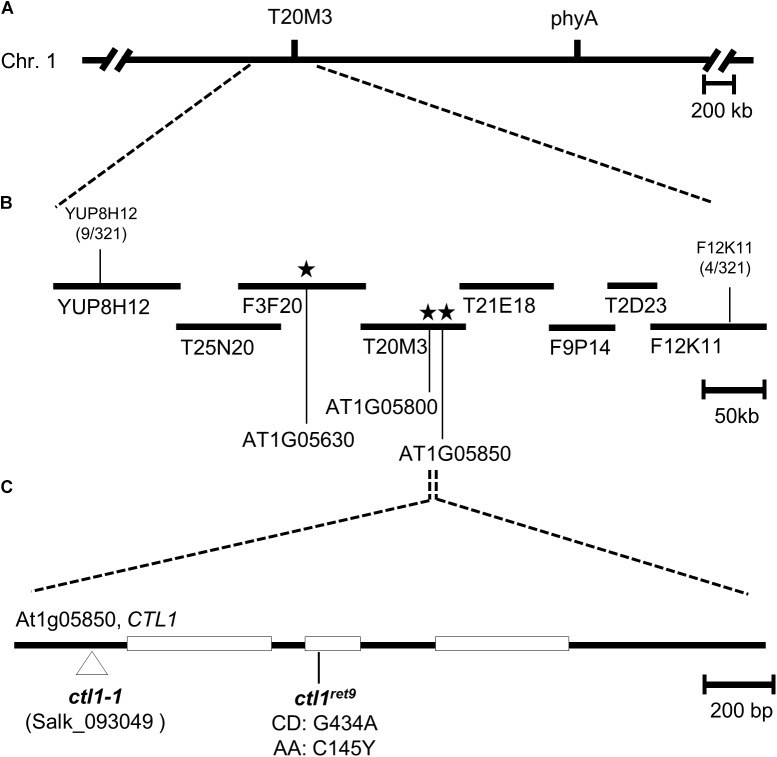
Positional cloning and molecular characterization of *ctl1^ret*9*^* mutation. **(A)** Genetic and physical maps of a 0.5-Mb region between the SSLP markers YUP8H12 and F12K11 in Arabidopsis chromosome 1 (Chr. 1). **(B)** BAC and TAC clones are depicted by filled bars, with marker positions labeled. Numbers bellow the markers indicate recombination events and chromosomal crosses. Asterisks indicate locations of putative candidate EMS mutations analyzed by the whole-genome sequencing with AGI annotation numbers At1g05630, At1g05800, and At1g05850. **(C)** The gene structure of *CTL1* (At1g05850): the protein-coding regions are shown as open boxes, and introns and untranslated regions are indicated as lines. The *ctl1^ret*9*^* mutation was a G to A mutation within the second exon, which changed the 145th codon from Cys (C) to Tyr (Y). The locations of mutations in *ctl1-1* (SALK _093049) and *ctl1^ret*9*^* are indicated.

*^a^The hypocotyl length of the 3-day-old etiolated seedlings with acsinone7030 treatment is twice longer than without (MS medium), and seedling hypocotyls in MS medium are shorter than those of Col-0. ^b^The calculated χ^2^ value was based on the expected ratio of 3:1 for individuals sensitive versus insensitive to acsinone7303, assuming that ctl1^ret9^ is a single recessive mutation; degree of freedom = 1. (P, probability). ^c^ret9 (ctl1^ret9^ eto1-4) was crossed with ctl1-1 for allelic analysis. The mutation is indicated in Figure 1A.*

**FIGURE 2:**
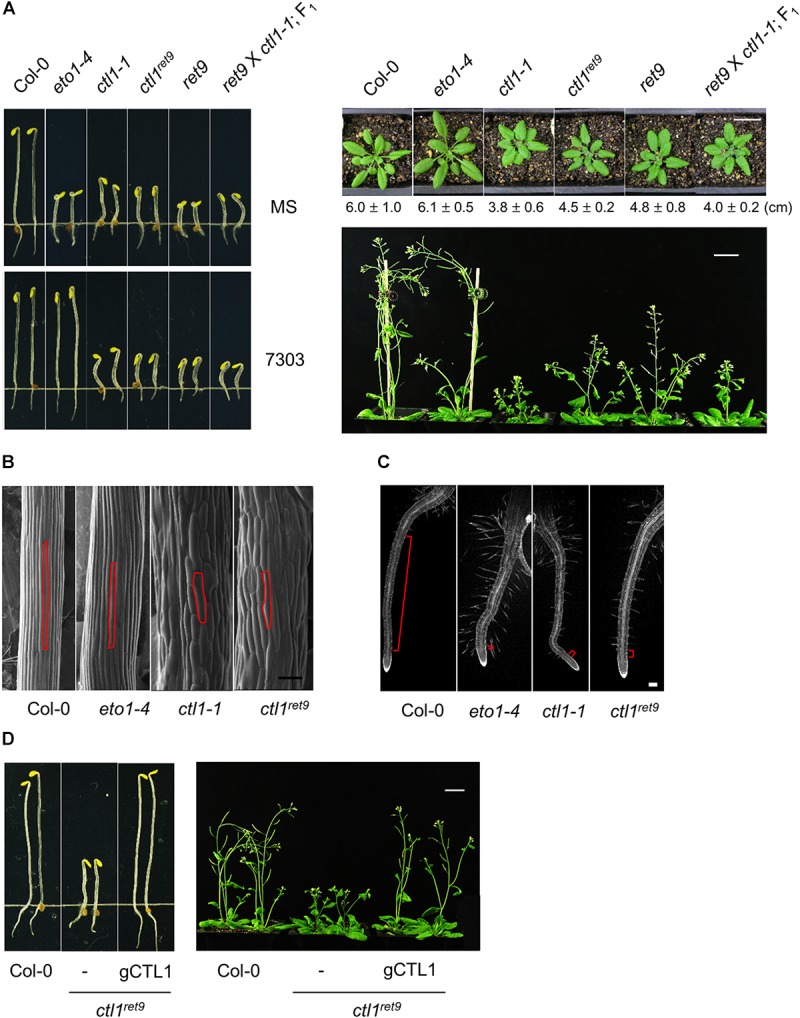
Phenotype and complementation analysis of Arabidopsis *ctl1^ret*9*^* mutant. **(A)** Left panel: phenotype of 3-day-old etiolated seedlings of Col-0 and mutants, including *eto1-4*, *ctl1-1*, *ctl1^ret*9*^*, and *ret9* (*eto1-4ctl1^ret*9*^*) and F1 progeny of *ret9* crossed to *ctl1-1*, treated without (MS) or with acsinone7303 (10 μM). Right panel: rosette plants of Col-0 and various mutants. Photos were taken when plants were 5 (top) or 7 (bottom) weeks old. Data of rosette diameters are shown as mean ± SD. Bar = 2 cm. **(B)** Scanning electron microscopy of hypocotyl epidermal cells from 3-day-old etiolated Col-0, *eto1-4*, *ctl1^ret*9*^*, and *ctl1-1* seedlings on MS medium, with one of the epidermal cells highlighted in red. Bar = 100 μm. **(C)** Confocal microscopy of roots from 3-day-old etiolated Col-0, *eto1-4*, *ctl1^ret*9*^*, and *ctl1-1* seedlings stained with propidium iodide (PI). Red lines highlight the elongation zone. Bar = 200 μm. **(D)** Complementation of *ctl1^ret*9*^* with a genomic clone of *CTL1.* The genomic construct containing the locus At1g05850 was introduced into *ctl1^ret*9*^* homozygous plants by *Agrobacterium*-mediated transformation. The phenotype of *ctl1^ret*9*^* was completely rescued in the presence of a genomic sequence of *CTL1* (*gCTL1*) in etiolated seedlings (left panel) and 6-week-old rosette plants (right panel). Bars = 2 cm.

A rough mapping result from 321 F_2_ progeny revealed that *ret9* was located at the top arm of chromosome 1 in the region between markers YUP8H12 and F12K11 (Figure 1A). To identify the causal mutation in *ret9*, we used pooled genomic DNA from 40 F_2_ seedlings for whole-genome sequencing by an Illumina sequencer. By comparing with the reference sequence of Col-0 between YUP8H12 and F12K11, we found 3 single-amino acid changes in *ret9*, including Trp639 to stop, Trp375 to stop and Cys145 to Tyr in the coding regions of genes annotated as At1g05630, At1g05800, and At1g05850, respectively (Figure 1B). Two approaches were used to find the causal mutation in *ret9*. First, we obtained T-DNA insertion mutants, SALK_081991, SAIL_221_B07, and SALK_093049, representing null alleles for each of the three loci. Only the null mutation (SALK_093049; hereafter *ctl1-1*) in At1g05850 had a highly similar phenotype to *ret9*, with reduced length in hypocotyls and roots of etiolated seedlings (Figure 2A, left panel). The At1g05850 locus encodes an endochitinase-like protein in Arabidopsis, CTL1 (Zhong et al., 2002).

Next, we sequenced *CTL1* in the *ret9* mutant and verified a G434A mutation at the second exon. The G-to-A substitution resulted in a missense mutation changing the 145th amino acid from Cys to Tyr (Figure 1C, C145Y). This substitution occurred in a highly conserved residue within the putative catalytic domain of CTL1 (Supplementary Figure S2). To further confirm that the G434A mutation in *CTL1* is responsible for the *ret9* phenotype, we performed an allelic analysis by crossing *ret9* with *ctl1-1* (SALK_093049) (Sanchez-Rodriguez et al., 2012) and examined the phenotype of F1 seedlings. All etiolated seedlings from the F1 generation of the cross (*ret9* × *ctl1-1*) were insensitive to acsinone7303, showing short hypocotyls and roots (Table 1). The F1 plants also had the small rosette leaves and semi-dwarfism of the *ctl1-1* mutant (Figure 2A, right panel). In addition, the rosette plants of *ret9* were smaller than those of *eto1-4*, with a small and compact size of rosette leaves and short petioles and reduced plant height. The allelic analysis revealed that the *ret9* phenotype was due to a substitution mutation in *CTL1* leading to the mutated protein CTL1^C145Y^, which is encoded by a new allele designated *ctl1^ret*9*^* (Table 1 and Figure 2A).

### Etiolated Seedlings of *ctl1^ret*9*^* Exhibit an Enhanced Triple Response in an *eto1* Genetic Background

The etiolated seedlings of *ret9* showed an enhanced phenotype when compared with *eto1-4*, which is reminiscent of the triple response phenotype including shortened and swollen hypocotyls, exaggerated apical hooks and short roots with excessive root hairs (Figure 2A, 3A). However, the hypocotyls remained short in *ret9* treated with acsinone7303, which differed from those of *eto1-4* with the same treatment (Figure 2A, left panel). To determine the roles of *eto1* and *ctl1* in the *ret9* phenotype, we isolated *ctl1^ret*9*^* via a genetic cross between Col-0 and *ret9*, which generated isogenic progeny, including the wild type (WT), *eto1-4*, *ctl1^ret*9*^*, and *ret9*, for further characterization. Without chemical treatment, 3-day-old etiolated seedlings of *ctl1^ret*9*^, eto1-4*, *ctl1-1*, and *ret9* exhibited short hypocotyls and roots (Figure 2A; left-top panel). When acsinone7303 was applied, the hypocotyls of etiolated *ctl1^ret*9*^* and *ctl1-1*, but not *eto1-4*, remained short (Figure 2A, left-bottom panel). Both *ctl1^ret*9*^* and *ret9* mutants showed reduced size of rosette leaves and plant height as compared with the WT and *eto1-4* (Figure 2A; right panel). The rosette diameter of *ctl1^ret*9*^* and *ret9* plants are approximately 75 and 78% of that in the WT and *eto1-4*, respectively, which is consistent with a previous observation (Zhong et al., 2002). On electron microscopy of epidermal tissues in hypocotyls of etiolated seedlings, *ctl1-1* and *ctl1^ret*9*^* had more swollen and shorter cells than the WT and *eto1-4* (Figure 2B). Microscopy also revealed increased root hairs (Supplementary Figure S3) and an extremely short elongation zone in roots of *eto1-4*, *ctl1-1*, and *ctl1^ret*9*^* (Figure 2C). These results suggest that the enhanced phenotype in root shortening of etiolated *ret9* was likely contributed by both *eto1-4* and *ctl1^ret*9*^*. However, *ctl1* and *eto1* may have a distinct role in cell elongation. We further performed a complementation analysis to confirm the association of *ctl1* mutation with the phenotype in *ctl1^ret*9*^.* A 2199-bp genomic DNA fragment containing *CTL1* was introduced into *ctl1^ret*9*^*: transgenic plants were indistinguishable from the WT in both etiolated seedlings and rosette plants (Figure 2D). Thus, our results support that *ctl1^ret*9*^* is the causal factor for the *ret9* phenotype.

**FIGURE 3:**
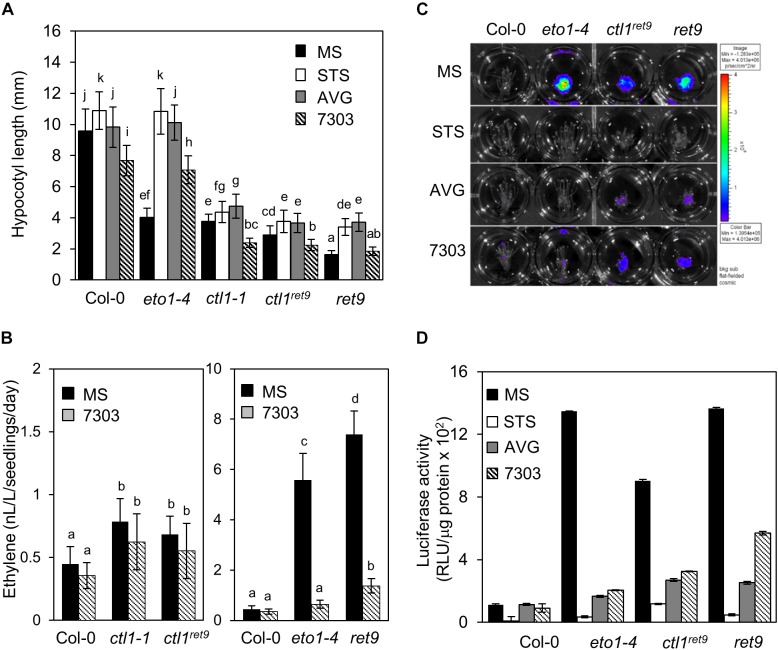
Ethylene production and response in etiolated *ret9* seedlings are suppressed by acsinone7303. **(A)** Hypocotyl length of 3-day-old etiolated seedlings of Col-0 and various mutants, including *eto1-4*, *ctl1-1*, *ctl1^ret*9*^*, and *ret9*, in the absence (MS) or presence of chemicals as indicated: AVG (5 μM), STS (10 μM), or acsinone7303 (10 μM). Data are mean ± SD of at least 25 seedlings for each treatment (*n* ≥ 25). A representative plot from 3 independent experiments is shown. **(B)** Ethylene levels of 3-day-old etiolated seedlings of Col-0 and mutants treated without (MS) or with acsinone7303 as indicated. Data are mean ± SD of 3 biological replicates. **(C)** Images of 3-day-old etiolated seedlings for luciferase activity and **(D)** quantification of *5xEBS*::*LUC* luciferase activity in Col-0, *eto1-4*, *ctl1^ret*9*^*, and *ret9*, containing p35Smin-*5xEBS*::*LUC*, treated without (MS) or with AVG (5 μM), STS (10 μM) and acsinone7303 (10 μM). The superimposed pseudocolor represents the photons emitted by the live cells after spraying luciferin (2 mM); the color scale bar on the right in **(C)** shows the photon counts (photon/s/cm^2^). Luciferase activity is measured as relative light unit (RLU) per μg of total protein. Data are mean ± SD of triplicate experiments. Different lowercase letters indicate statistical significance based on ANOVA with Duncan *post hoc* test (*p* < 0.05).

To determine whether the hypocotyl phenotype in etiolated *ctl1^ret*9*^* seedlings was regulated by ethylene biosynthesis and/or signaling, we measured the hypocotyl length of etiolated seedlings treated with specific inhibitors of ethylene perception [silver thiosulfate (STS)] or biosynthesis [acsinone7303 and aminoethoxyvinylglycine (AVG)]. The hypocotyl length of etiolated *ctl1-1* and *ctl1^ret*9*^* was slightly increased by STS and AVG, but the hypocotyl phenotype in etiolated *eto1-4* was completely suppressed and indistinguishable from that of the WT (Figure 3A and Supplementary Table S1). In addition, the hypocotyls of *ret9* treated with STS or AVG were elongated to the same degree as those of *ctl1-1* and *ctl1^ret*9*^*, which suggests that the hypocotyl phenotype of etiolated *ret9* seedlings may only partially depend on ethylene. We next measured ethylene level in *ctl1^ret*9*^*. Consistent with a previous observation (Zhong et al., 2002), ethylene level in etiolated *ctl1-1* and *ctl1^ret*9*^* seedlings was 2.0- and 1.5-fold that of the WT (Figure 3B, left panel and Supplementary Table S2). Ethylene level was higher in *ret9* than *eto1-4* even in the presence of acsinone7303 (Figure 3B, right panel and Supplementary Table S2), which suggests that *ctl1^ret*9*^* contributes to ethylene biosynthesis independent or downstream of ACS activity.

Next, we examined the ethylene response in *ctl1* mutants by measuring luciferase activity of transgenic plants containing a reporter gene responsive to ethylene with 5 copies of *EIN3* binding sequence (EBS) fused to a luciferase gene (*5xEBS::LUC*). As compared with the WT, in etiolated *eto1-4* seedlings, luciferase activity was highly induced and was abolished by acsinone7303, AVG, and STS (Figure 3C,D). Luciferase activities in *ctl1^ret*9*^* and *ret9* were fully suppressed by STS but only partially by AVG and acsinone7303 (Figure 3C,D). We noted that *ctl1^ret*9*^* induced a substantially high ethylene response in the absence of *eto1-4*. These observations suggest that the enhanced ethylene response and phenotype in *ret9* requires ethylene perception and biosynthesis resulting from a combined effect of both *eto1-4* and *ctl1^ret*9*^*. Furthermore, *CTL1* may be involved in modulating ACO activity or ethylene sensitivity or both.

### Etiolated *ctl1* Mutants Showed Stunted Root Growth and Altered Response to the Immediate Precursor of Ethylene, ACC

**FIGURE 4:**
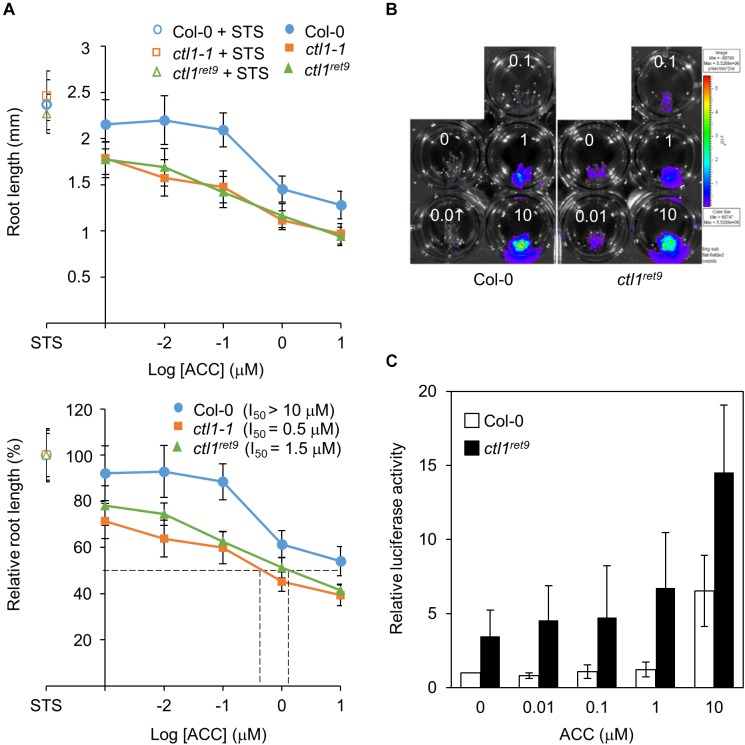
Etiolated *ctl1^ret*9*^* seedlings show enhanced sensitivity to ACC in primary root elongation. **(A)** ACC dose-response analysis of Col-0 and *ctl1* mutants. Upper panel: actual root length in 3-day-old etiolated seedlings of Col-0 (circle), *ctl1^ret*9*^* (triangle) and *ctl1-1* (square) treated with STS (open symbols, 10 μM) or AVG (filled symbols, 5 μM) supplemented with different concentrations of ACC in log scale ranging from 0 to 10 μM on the *x*-axis. Lower panel: relative inhibition of root length (root length at 10 μM STS set to 100%). The concentrations of ACC causing 50% inhibition of root length (I_50_) are denoted by dashed lines. Data are mean ± SD of at least 25 measurements for each treatment (*n* ≥ 25). A representative plot from 1 of 3 independent experiments is shown. **(B)** Images of 3-day-old etiolated seedlings for luciferase activity and **(C)** quantification of *5xEBS*::*LUC* activity in Col-0 and *ctl1^ret*9*^*, both containing *p35Smin*-*5xEBS*::*LUC*, treated without (MS) or with AVG (5 μM) supplemented with different concentrations of ACC (0 to 10 μM). The superimposed pseudocolor represents the photons emitted by the live cells after luciferin treatment (2 mM); the color scale bar on the right shows the photon counts (photon/s/cm^2^). Luciferase activity is measured as relative light unit (RLU) per μg of total protein. Data are mean ± SD of three biological replicates. The fold change in luciferase activity with each treatment is compared with that in Col-0 without ACC, set to 1.

To investigate whether enhanced ethylene sensitivity is attributed to the phenotype of *ctl1^ret*9*^*, we analyzed the dose-dependent response to ACC in the roots of etiolated seedlings. Endogenous ACC biosynthesis was suppressed by the competitive inhibitor of ACS enzyme, AVG, in the following experiments. In the presence of STS, the root lengths of *ctl1-1* and *ctl1^ret*9*^* were nearly indistinguishable from those of the WT (Figure 4A, +STS, open symbols, top plot, *p* > 0.05). However, the roots of *ctl1-1* and *ctl1^ret*9*^* were shorter than those of the WT without STS or ACC but were responsive to ACC, similar to the WT.

We next analyzed the effect of ACC on root elongation by measuring relative root length in etiolated seedlings treated with different concentrations of ACC as described previously (Cancel and Larsen, 2002). With increasing concentrations of ACC, both *ctl1-1* and *ctl1^ret*9*^* showed greater sensitivity than the WT in primary root elongation (Figure 4A, bottom plot). An amount of 10 μM of ACC was required to achieve approximately 50% inhibition of primary root growth in WT seedlings (I_50_ > 10 μM), significantly higher than that for *ctl1-1* (I_50_ = 0.5 μM) and *ctl1^ret*9*^* (I_50_ = 1.5 μM). The responsiveness to ACC was also examined for hypocotyls of *ctl1* mutants. Unlike roots, the hypocotyls of *ctl1-1* and *ctl1^ret*9*^* were significantly shorter than those of the WT in the presence of STS or ACC (Supplementary Figure S4), which suggests that the stunted hypocotyl phenotype in *ctl1* mutants is largely independent of ethylene. Collectively, these results indicate that etiolated seedlings of *ctl1* mutants are sensitive to the immediate precursor of ethylene, ACC for inhibition of root growth.

We further analyzed the ethylene response of *ctl1* mutants by measuring luciferase activity in the presence of various concentrations of ACC combined with AVG to suppress endogenous ACC biosynthesis. In WT seedlings, the *5xEBS::LUC* transgene was activated on exposure to ACC, whereas that in *ctl1^ret*9*^* showed enhanced sensitivity to ACC dose-dependently, starting at low concentrations of exogenous ACC that did not evoke reporter activity in the WT (Figure 4B,C). The luciferase reporter gene in *ctl1^ret*9*^* was constitutively active, with 2.8-fold higher activity than in the WT without exogenous ACC and was increased by 23% in *ctl1^ret*9*^* at 0.01 μM ACC (Figure 4C). Furthermore, 10 μM was required for the WT to show the same level of luciferase activity as 1 μM ACC in *ctl1^ret*9*^*. Collectively, our data suggest that *ctl1^ret*9*^* shows enhanced sensitivity to ACC in etiolated seedlings, which may account for the suppressed elongation of roots and hypocotyls.

### Phenotypic Analyses of *ctl1^ret*9*^* Combined With Ethylene-Insensitive Mutants

**FIGURE 5:**
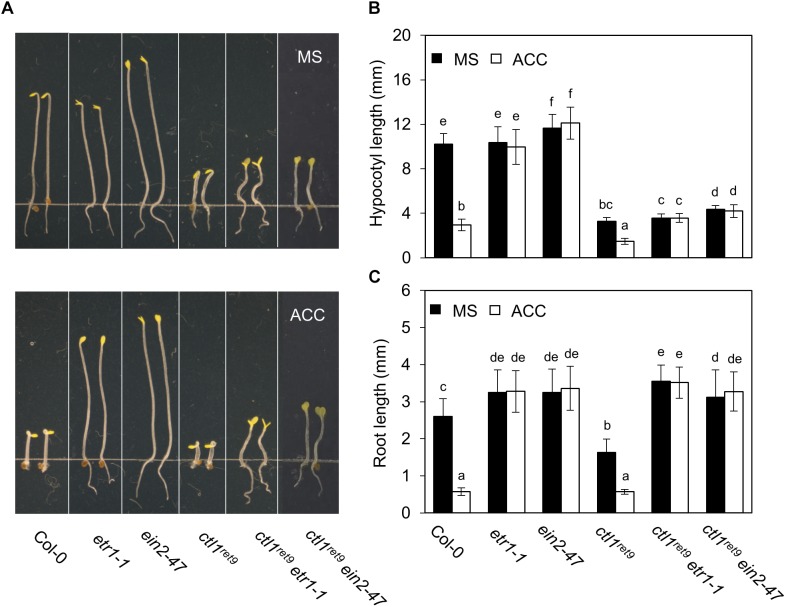
Ethylene response is required for the root phenotype in *ctl1^ret*9*^* mutant. **(A)** The phenotype of 3-day-old etiolated seedlings of Col-0 and mutants, including *etr1-1*, *ein2-4*, *ctl1^ret*9*^*, *ctl1^ret*9*^etr1-1*, and *ctl1^ret*9*^ein2-47*, on MS medium supplemented without (MS) or with ACC (10 μM). **(B)** Quantification of hypocotyl and **(C)** root length of 3-day-old etiolated seedlings of mutants without (MS) or with ACC (10 μM). Data are mean ± SD of at least 25 seedlings for each treatment (*n* ≥ 25). A representative plot from 1 of 3 independent experiments is shown. Different lowercase letters indicate statistical significance based on ANOVA with Duncan *post hoc* test (*p* < 0.05).

To investigate the role of ethylene response in CTL1 function, we introduced the *ctl1^ret*9*^* allele in known ethylene insensitive mutants, *etr1-1*, *ein2-47*, and *ein3-1eil1-1*, and examined hypocotyl and root phenotypes in etiolated seedlings (Figure 5A). Arabidopsis *etr1-1* bears a dominant mutation of the ethylene receptor *ETR1*, which fails to bind ethylene hormone and leads to ethylene insensitivity by constitutively activating CTR1 kinase (Chang et al., 1993). The *ein2-47* was isolated from a T-DNA insertion mutant (SALK_086500C) in this study and represents a complete loss-of-function allele of *EIN2*, which encodes a key positive regulator in the ethylene response pathway (Alonso et al., 1999). The roots of *ctl1^ret*9*^etr1-1* and *ctl1^ret*9*^ein2-47* were 2.2- and 1.9-fold longer than those of *ctl1^ret*9*^* (Figure 5C), whereas hypocotyl elongation in *ctl1^ret*9*^etr1-1* and *ctl1^ret*9*^ein2-47* remained inhibited, as in *ctl1^ret*9*^* (Figure 5B). Root lengths of *ctl1^ret*9*^etr1-1* and *ctl1^ret*9*^ein2-47* did not significantly differ from those of *etr1-1* or *ein2-47* (Figure 5C). When germinated in the dark and supplemented with ACC, both *ctl1^ret*9*^* and WT seedlings showed significantly reduced elongation of hypocotyls and roots (Figure 5B,C). However, only shortening of roots but not hypocotyls in *ctl1^ret*9*^* was suppressed by *etr1-1* or *ein2-47* to the same degree as in single ethylene insensitive mutants, which suggests that a functional ethylene response pathway is required for the root phenotype of *ctl1^ret*9*^* (Figure 5C). Moreover, the activity of a luciferase reporter gene dependent on EIN3/EIL1 in *ctl1^ret*9*^* was abolished by both *etr1-1* and *ein3-1eil1-1* with or without ACC (Supplementary Figure S5). These results are consistent with those by using STS to block ethylene perception (Figure 3C). Thus, suppressed root elongation in etiolated *ctl1^ret*9*^* seedlings mainly depends on an intact ethylene response pathway, and possibly an ethylene-independent mechanism accounts for CTL1-mediated hypocotyl elongation.

### Mutations in *CTL1* Induce Genes Encoding ACC Oxidase

**FIGURE 6:**
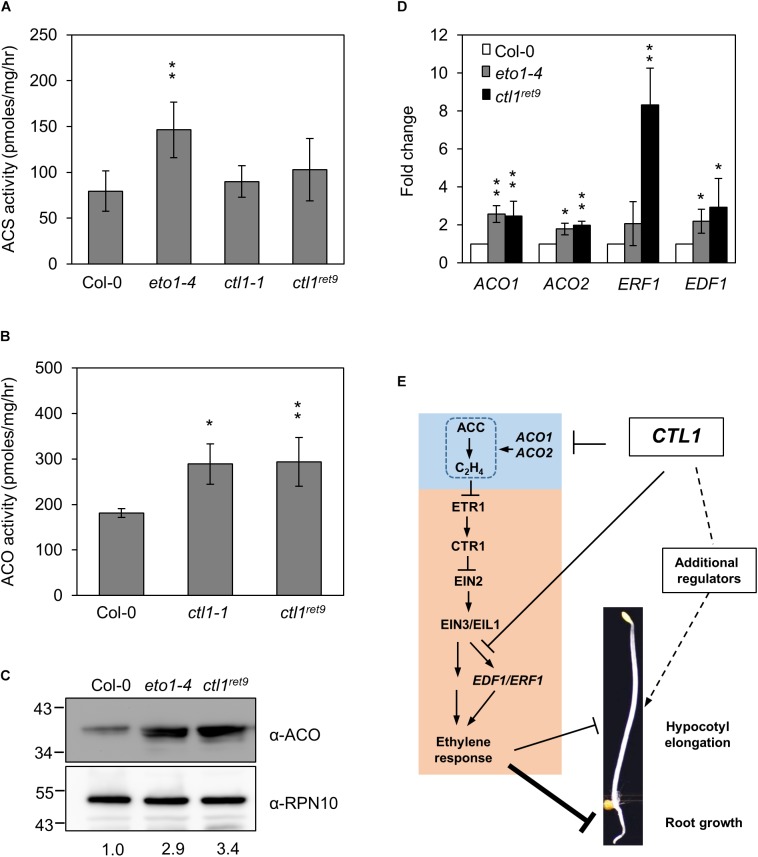
ACC oxidase activity is increased in *ctl1^ret*9*^* mutant. **(A)** The enzyme activity of ACS and **(B)** ACO is determined by the rate of ethylene production by converting SAM to ACC (for ACS) or ACC to ethylene (for ACO), respectively, with protein extracts from 3-day-old etiolated seedlings of Col-0, *eto1-4*, *ctl1-1*, and *ctl1^ret*9*^*. Data are mean ± SD of three biological replicates. Asterisks indicate a significant difference from Col-0 as determined by student’s *t*-test (^∗^*p* < 0.05 and ^∗∗^*p* < 0.01). **(C)** Western blot analysis of ACO protein levels in 3-day-old etiolated seedlings from Col-0, *eto1-4*, and *ctl1^ret*9*^*. ACO proteins were detected with an antibody against Arabidopsis ACO, and anti-RPN10 antibody was the loading control. The protein level of ACO in the wild type (Col-0) was arbitrarily set to 1. **(D)** Quantitative real-time RT-PCR analysis of representative genes responsive to ethylene, including *ACO1*, *ACO2*, *ERF1*, and *EDF1*, in Col-0, *eto1-4*, and *ctl1^ret*9*^*, from 3-day-old etiolated seedlings. Data are normalized to the expression of *UBQ10*, and the expression of each gene in Col-0 was set to 1. Data are mean ± SD of three biological replicates. Asterisks indicate significant differences from Col-0 as determined by student’s *t*-test (^∗^*p* < 0.05 and ^∗∗^*p* < 0.01). **(E)** A hypothetic model to illustrate how CTL1 regulates hypocotyl and root elongation in dark-grown Arabidopsis seedlings. CTL1 is proposed to maintain cell wall integrity by ordered cellulose deposition. The model highlights that loss-of-function mutations in *CTL1* result in reduced root elongation mainly dependent on ethylene by increasing ACO activity and expression of genes responsive to ethylene. Ethylene may have a minor role in the hypocotyl elongation mediated by CTL1, which likely involves other regulators responsive to cell wall integrity. Arrow and block symbols indicate activation and repression modes, respectively; solid lines indicate involvement of regulatory interactions without being necessarily a direct effect and dashed lines indicate unidentified regulatory pathways.

To determine whether ethylene biosynthesis is mis-regulated in *ctl1* mutants, we examined the expression and enzymatic activity of 2 key enzymes, ACS and ACO, in the ethylene biosynthetic pathway. As a control, the ACS activity in *eto1-4* was 1.9-fold higher than in the WT. In contrast, ACS activity did not differ between *ctl1-1* and *ctl1^ret*9*^* and the WT (Figure 6A). However, ACO activity was approximately 1.6-fold higher in both *ctl1^ret*9*^* and *ctl1-1* than the WT (Figure 6B), which suggests a causal factor for a higher basal level of ethylene in *ctl1* mutants. The protein level of ACO was 2.9- and 3.4-fold higher in *eto1-4* and *ctl1^ret*9*^* than the WT (Figure 6C). In addition, the mRNA expression of *ACO1* and *ACO2* was significantly upregulated in *eto1-4* and *ctl1^ret*9*^* (Figure 6D). The expression of *ACO1* and *ACO2* was 2.8- and 1.8-fold higher in *ctl1^ret*9*^* than the WT. Similarly, the expression of two direct target genes responsive to EIN3, *ETHYLENE RESPONSE FACTOR1* (ERF1) and *ETHYLENE RESPONSE DNA-BINDING FACTOR1* (*EDF1*) (Chao et al., 1997; Chang et al., 2013), was higher by 8.3- and 2.9-fold, respectively, in *ctl1^ret*9*^* than in the WT. Because ACO activity is directly and positively associated with ethylene production, and a positive feedback regulation of *ACO* gene expression has been documented (Nakatsuka et al., 1998), elevated ethylene level and enhanced ethylene sensitivity in *ctl1* mutants likely result from increased expression of ACO genes and those potentially involved in relaying the ethylene signal. Thus, the upregulated ethylene biosynthesis mediated by ACO activity in *ctl1* seedlings is attributed to the phenotype of roots and to some extent hypocotyls, which avoids inhibition of acsione7303 on ACS activity in the chemical genetics screen for Arabidopsis *ret* mutants.

## Discussion

By using a phenotype-based chemical screening, we identified small-molecule compounds suppressing the ethylene phenotype in etiolated *eto1* seedlings. Acsinones are a group of chemical compounds that act as uncompetitive inhibitors of ACS and are distinct from AVG in chemical structure and mode of action. To explore additional roles of acsinones, we use a genetic approach to screen for Arabidopsis mutants with reduced sensitivity to acsinones (Chen et al., 2013). The *ret9* mutant was isolated with such a screen and exhibited a constitutive triple response phenotype resembling etiolated *eto1-4* seedlings without acsinone7303 (Figure 2A). Positional cloning and whole-genome sequencing revealed that *ret9* (*eto1-4ctl1^ret*9*^*) carried a missense mutation in Arabidopsis *CTL1*, which is involved in cell wall integrity and abiotic stress response. Further characterization showed that the *ctl1^ret*9*^* mutation phenocopied a T-DNA insertional mutant, *ctl1-1* (Figure 2), which suggests that *ctl1^ret*9*^* is a new loss-of-function allele in *CTL1*. In line with previous findings, 2 different alleles of *ctl1* produce a moderate yet significant level of ethylene (Figure 3B). The hypocotyls of etiolated *ctl1^ret*9*^* seedlings are shorter than those of the WT, which is independent of the *eto1-4* allele. Consistently, inhibitors of ethylene biosynthesis (ascinone7303 and AVG) and perception (STS) effectively suppressed the hypocotyl phenotype, ethylene emission and ethylene-responsive reporter gene expression of etiolated *eto1-4* seedlings but not *ret9* and *ctl1* mutants (Figure 3). In addition, *ctl1^ret*9*^* showed enhanced ethylene sensitivity under conditions with no apparent ethylene response in the WT (Figure 3, 4). The etiolated *ctl1* seedlings were highly sensitive to ACC and dose-dependently in suppressing primary root elongation (Figure 4A). Genetic analysis of combination ethylene-insensitive mutants indicated a requirement for the ethylene-response pathway for *CTL1* in the phenotype of primary root but not hypocotyl elongation (Figure 5). The transcript and protein levels of ACO genes were increased in *ctl1^ret*9*^* and *ctl1-1*. Thus, moderately elevated ethylene production in *ctl1* was modulated by ACO but not ACS activity (Figure 6). Furthermore, 2 of the well-characterized ethylene responsive genes, *ERF1* and *EDF1*, were upregulated in *ctl1^ret*9*^* as well as *eto1-4* (Figure 6D).

Based on the results in this study, we propose a working model diagrammed in Figure 6E to provide a functional connection between CTL1 and ethylene hormone to regulate development of etiolated Arabidopsis seedlings. Loss-of-function mutations in *CTL1* lead to upregulation of *ACO1* and *ACO2* to promote moderate ethylene production and some of the ethylene-responsive genes, such as *EDF1* and *ERF1*, for potentially enhanced ethylene response. Ethylene response pathway plays a major role in the root architecture of etiolated seedlings dependent on *CTL1*. However, additional regulators are likely involved in the development of apical hook and hypocotyl growth, of which JA may have a role in responding to cell wall rigidity. This study provides new information for how maintenance of cell wall integrity mediated by CTL1 may affect seedling development by modulating ethylene function.

### Ethylene Response and Cell Wall Integrity in *ctl1* Mutant

The seedling phenotype of *ctl1* shows characteristics of mutants defective in cell wall integrity due to perturbed cellulose synthesis. Mutants defective in cellulose synthesis frequently show a phenotype of reduced elongation, radial swelling and lignin deposition in roots (Cano-Delgado et al., 2003; Tsang et al., 2011). Results from studies of cell wall-deficient mutants or use of isoxaben, a cellulose synthesis inhibitor, suggest a functional connection between cell wall modifications and the ethylene biosynthesis and/or response pathway. ACC is the immediate precursor of ethylene that mediates a rapid reduction in root elongation in 4-day-old light-grown seedlings in response to the short-term cell wall damage induced by isoxaben through an ethylene-independent pathway (Tsang et al., 2011). Similarly, perturbed cell wall integrity caused by boron deficiency leads to a rapid inhibition of root elongation, which is mediated by ethylene, auxin, and reactive oxygen species (ROS)-dependent pathways (Camacho-Cristobal et al., 2015).

Mutations in the Arabidopsis receptor-like kinases (RLKs) *FEI1* and *FEI2* conferred short and swollen roots when plants were grown on agar medium containing 4.5% sucrose (Xu et al., 2008). The *fei1fei2* mutant fails to sense cell wall integrity and exhibits a conditionally anisotropic growth phenotype in roots. Although *fei1fei2* produced ethylene at a basal level as in the WT, the swollen root phenotype could be suppressed by α-aminoisobutyric acid and aminooxyacetic acid, which inhibit ACO and ACS activity, respectively. Both FEI1 and FEI2 physically interact with ACS5 and ACS9, with no change in protein level and activity of ACS enzymes in the *fei1fei2* mutant. This observation suggests that FEI1 and FEI2 may play a role in cell wall architecture dependent on ACC rather than ethylene (Xu et al., 2008; Tsang et al., 2011). Unlike the *fei1fei2* mutant, in *ctl1* seedlings, the root phenotype depends on the ethylene response because the stunted root morphology can be reversed by *etr1* and *ein2*. Moreover, the plasma membrane-localized RLK *FERONIA (FER)* is also considered a cell wall integrity sensor that relays a brassinosteroid signal to antagonize the effect of ethylene on hypocotyl growth. The Arabidopsis null *fer* mutant shows a slight increase in ethylene level and sensitivity to exogenous ethylene (Deslauriers and Larsen, 2010). FER directly interacts with SAM synthetase and reduces SAM production and then ethylene biosynthesis (Mao et al., 2015). In *ctl1* mutant, the elevated ethylene production was not due to changes of SAM synthetase nor ACS activity, but induction of ACO genes, which provide an additional node in ethylene pathway responding to cell wall integrity in Arabidopsis. Together, these findings imply that ethylene or its precursor ACC is involved in the structural or compositional alterations of the cell wall resulting from defective RLKs via distinct mechanisms.

Ectopic lignification in non-lignified cells is critical for plants in response to perturbed cell wall integrity (Cano-Delgado et al., 2003; Hematy et al., 2007). Some of the aberrant secondary growth in cell wall-deficient mutants involves increased ethylene level. When mung bean sprouts are treated with ethephon, an ethylene-releasing chemical, primary root elongation is inhibited and lignification is enhanced in roots dose-dependently (Huang et al., 2013). In addition, overexpression of the Arabidopsis auxin biosynthetic genes *YUCCA8* and *YUCCA9* led to substantially increased lignification in plant aerial tissues, accompanied by elevated transcript levels of a number of genes involved in ethylene biosynthesis and response (Hentrich et al., 2013). Collectively, these and our findings suggest that ethylene may have an executive role in responding to cell wall damage, followed by changing the root morphology and lignification of the cell wall.

### Mutations in *CTL1* Show Enhanced Root-Specific Sensitivity to Ethylene in Etiolated Seedlings

Ethylene is functionally relevant to the root phenotype in different alleles of the *ctl1* mutant. Some of the root characteristics in the *ctl1/arm* mutant, such as root swelling, root hair proliferation and shortened primary roots, are similar to those in ethylene-overproducer (*eto*) mutants under high nitrate condition (Hermans et al., 2011). Blocking the ethylene production or response suppresses the phenotype of exaggerated hook curvature and excessive root hairs in etiolated *ctl1/elp1* seedlings (Zhong et al., 2002; Hermans et al., 2011). In addition, root radial swelling and increased root hair density of light-grown *ctl1/arm* seedlings is reversed by the antagonism of the ethylene receptor under high nitrate conditions (Zhong et al., 2002; Hermans et al., 2011). These observations suggest a causal role of ethylene in the seedling phenotype of different *ctl1* alleles. The *ctl1^ret*9*^* mutant showed enhanced sensitivity to exogenous ACC and dose-dependently in inhibiting primary root elongation (Figure 4). ACC induces a short-root and exaggerated-hook phenotype in etiolated *ctl1^ret*9*^* seedlings as in the WT, which can be suppressed by *etr1-1* and *ein2-47* (Figure 5A). Thus, the altered apical hook and root elongation in *ctl1^ret*9*^* is ethylene-dependent instead of a general defect in cell expansion. The formation of an apical hook and inhibition of primary root growth are regulated by biosynthesis and asymmetric accumulation of auxin, which is modulated by ethylene (Mazzella et al., 2014). Jasmonic acid (JA) acts synergistically with ethylene in root hair development but antagonistically in apical hook formation by repressing *HOOKLESS1* (*HLS1*) expression (Zhu et al., 2011; Song et al., 2014). Interestingly, we found that *ctl1^ret*9*^etr1-1* displayed unfolding cotyledons, which are much more enhanced than those of *etr1-1* (Figure 5A). The pronounced cotyledon opening phenotype in *ctl1^ret*9*^etr1-1* mimics that in etiolated seedlings treated with JA (Zheng et al., 2017), which may be due to absence of ethylene response in *ctl1^ret*9*^etr1-1* to antagonize JA. Thus, JA may have a role in *ctl1* seedling phenotype. However, the mechanism underlying how the function of cell wall-localized CTL1 is linked to the ethylene or other hormones to regulate root elongation and apical-hook formation is not completely clear.

Defects in cellulose synthesis in the plant cell wall may invoke the ethylene and JA function to enhance resistance to pathogens, alter cell wall structure and composition, and produce ectopic deposition of lignin (Ellis et al., 2002; Cano-Delgado et al., 2003; Hamann et al., 2009). The Arabidopsis *constitutive expression of vsp1* (*cev1*) mutant, which is allelic to *cesa3*, shows increased production of JA and ethylene and consequently increased expression of stress-responsive genes, such as *PDF1.2* (*PLANT DEFENSIN1.2*) and *BASIC CHITINASE* (*ChiB*). The shortened hypocotyl of etiolated *cev1* seedlings was suppressed by *etr1*, which suggests that the phenotype depends on ethylene response (Ellis and Turner, 2001; Ellis et al., 2002). In contrast, the hypocotyl of etiolated *ctl1* seedlings was less sensitive to exogenous ACC (Supplementary Figure S4). The hypocotyls remained short in *ctl1^ret*9*^* on treatment with ethylene inhibitors and by a genetic cross with ethylene-insensitive mutants (Figure 3, 5).

Our results suggest that ethylene may not play a major role in the aberrant hypocotyl phenotype of etiolated *ctl1^ret*9*^* seedlings, which is likely regulated by other mechanisms. JA inhibits hypocotyl elongation in etiolated Arabidopsis seedlings by suppressing CONSTITUTIVE PHOTOMORPHOGENESIS 1 (COP1) function, which is critical for the maintenance of skotomorphogenesis (Zheng et al., 2017). Based on transcriptome analysis, the inhibition of hypocotyl growth by JA is likely due to regulate gene expression involved in cell wall organization, growth and auxin responses. In addition, the Arabidopsis RLK encoded by *THESEUS1* (*THE1*) senses cell wall perturbations to regulate cell expansion. THE1 is required for generation of cell wall damage-induced ROS to control lignin production in the root elongation zone (Denness et al., 2011). Despite an unclear direct functional link between the cell wall integrity sensor and CTL1, the *the1-3* mutation partially suppresses the reduced hypocotyl elongation in several cellulose-deficient mutants including *cesa1, cesa3, korrigan* (*kor*) and *ctl1/pom1* itself (Hematy et al., 2007). Collectively, our work suggests that ethylene plays a major role in reducing root elongation of *ctl1* mutants, while additional regulators such as JA hormone and/or THE1 are involved in CTL1-dependent hypocotyl growth in etiolated seedlings.

### The Enhanced Ethylene Sensitivity in *ctl1* Is Likely Due to Increased Expression of Genes Involved in Ethylene Biosynthesis and Response

The *ctl1* mutants produce more ethylene than does the WT when germinated in the dark (Figure 3) (Zhong et al., 2002; Hermans et al., 2011). Antagonism of the ethylene receptor but not ACS inhibitors significantly reduced the activity of a luciferase reporter gene mediated by EIN3/EIL1. By using activity assays and quantitative measurements of transcript and protein levels, we confirmed that increased ACO activity is the causal factor for elevated ethylene production and sensitivity in etiolated *ctl1^ret*9*^* seedlings. The *BRITTLE CULM15* (*BC15/OsCTL1*) encodes a chitinase-like protein required for cellulose synthesis and cell wall remodeling in rice (*Oryza sativa*). The *bc15* mutant shows reduced cellulose content and mechanical strength in the cell wall. In line with our findings, the expression of *ACO* (Os05g05670) was found upregulated in *bc15* on whole-genome RNA-seq analysis (Wu et al., 2012). Although ACS is generally considered the rate-limiting step in ethylene production (Yang and Hoffman, 1984), the activity of *ACC* oxidase is another major regulator in ethylene biosynthesis responding to developmental and environmental signals. Ethylene production during seed germination is associated with increased ACO activity at the transcriptional level (Matilla and Matilla-Vazquez, 2008; Linkies and Leubner-Metzger, 2012). Recently, it has been shown that Arabidopsis *ACO1* is predominantly expressed in roots and cotyledons but not hypocotyls in dark-grown seedlings (Park et al., 2018). The tissue-specific expression of *ACO1* is in line with our result that ethylene function accounts for the root growth phenotype in etiolated *ctl1* seedlings.

CTL1 may also modulate ethylene sensitivity in plants. The *arm/ctl1* seedlings show reduced elongation of primary roots, radical swelling, increased lateral roots and root hairs responding to high concentrations of nitrate. Intriguingly, although the ethylene emanation in etiolated *arm* seedlings is 50% lower under high nitrate (60 mM NO_3_^-^) than low nitrate (0.6 mM NO_3_^-^), the ethylene sensitivity in the *arm* mutant is enhanced by elevated nitrate concentration (Hermans et al., 2011). Both the *ctl1^ret*9*^* and the null mutant *ctl1-1* are sensitive to exogenous ACC and dose-dependently in inhibiting primary root elongation (Figure 4A). Consistently, we found higher ethylene sensitivity in *ctl1^ret*9*^* than the WT on reporter gene analysis (Figure 4B,C). The expression of 2 representative genes responsive to ethylene, *ERF1* and *EDF1*, was significantly higher in *ctl1^ret*9*^* than in *eto1-4* and the WT (Figure 6D). The transcript levels of *ERF1* were 8.2- and 4-fold higher in *ctl1^ret*9*^* than Col-0 and *eto1-4*, respectively. This finding is interesting yet intriguing because ethylene level was not proportionally higher in etiolated *ctl1* than *eto1-4* seedlings (Figure 3B), which suggests that ethylene sensitivity is likely enhanced by *ctl1^ret*9*^* or additional regulators are involved in CTL1-dependent gene regulation. One of the potential regulators is JA. It has been shown that JA and ethylene co-regulate *ERF1* expression by a mechanism that JA alleviates the suppression of EIN3 transcriptional activity by JA-ZIM domain (JAZ) proteins (Zhu et al., 2011). The possible role of JA in *ctl1* phenotype can be revealed by using JA response mutants in the *ctl1* background for genetic analysis.

Transcriptome analysis in the rice *bc15* mutant showed the expression of several *AP2/ERF* genes significantly increased (Wu et al., 2012). Overexpression of *ERF1* in Arabidopsis seedlings reduced primary root growth in the dark (Mao et al., 2016). ERF1 directly regulates *ANTHRANILATE SYNTHASE α1* that results in auxin accumulation and ethylene-induced inhibition of root growth in Arabidopsis (Swarup et al., 2007;Mao et al., 2016). In line with these observations, we found that dark-grown *ctl1* seedlings showed a shorter elongation zone in roots than did the WT, possibly due to an increased local concentration of auxin (Figure 2C). Taken together, our results suggest that the enhanced sensitivity to ethylene in *ctl1* may involve a combined effect of elevated ACO activity and increased expression of some if not all of the ethylene-responsive genes. The next task is to show how ethylene perception and sensitivity could be regulated by *CTL1*.

In summary, we isolated a new allele of *CTL1*, *ctl1^ret*9*^*, and demonstrated that *CTL1* plays a role in altering ethylene biosynthesis and sensitivity. Mutations in *CTL1* enhance the transcript level of several ethylene-responsive factors, which are related to control of the magnitude of ethylene response and biosynthesis. Genetic analysis of *ctl1^ret*9*^* combined with ethylene-insensitive mutants showed that both CTL1 and ethylene are involved in modulating the root architecture of seedlings, which reveals a functional role of ethylene in cellulose-deficient mutants and/or cell wall rigidity in response to abiotic stress.

## Author Contributions

W-SL, L-CW, and S-YG designed experiments. L-CW, S-YG, and C-MC performed the experiments. W-SL, L-CW, and S-YG analyzed the data and wrote the manuscript. W-SL and L-CW provided supervision, funding, and reagents.

## Conflict of Interest Statement

The authors declare that the research was conducted in the absence of any commercial or financial relationships that could be construed as a potential conflict of interest.

## Footnotes

1 http://rsbweb.nih.gov/ij/

2 http://www.arabidopsis.org/browse/Cereon/index.jsp

3 http://www.onetry.cn/

4 http://www.arabidopsis.org/browse/Cereon/index.jsp

## Abbreviations

ACC: 1-aminocyclopropane-1-carboxylic acid
ACO: ACC oxidase
ACS: ACC synthase
Acsinone7303: ACS inhibitor quinazolinone 7303
CTL1: CHITINASE LIKE1
ETO1: ETHYLENE OVERPRODUCER1
*ret*: *revert to eto1*
