# Supplementary material for: CHITINASE LIKE1 Regulates Root Development of Dark-Grown Seedlings by Modulating Ethylene Biosynthesis in Arabidopsis thaliana
Source: Front Plant Sci. 2019 May 14;10:600. doi: 10.3389/fpls.2019.00600 (PMC6530356; doi:10.3389/fpls.2019.00600)
Supplement: Supplementary file 1 [file Data_Sheet_1.PDF]

Supporting Information

CHITINASE LIKE1 Regulates Root Development of Dark-grown Seedlings by Modulating Ethylene Biosynthesis in *Arabidopsis thaliana*

Shin-Yuan Gu[a,b,#], Long-Chi Wang[c,#], Chiao-Mei Cheuh[a], and Wan-Sheng Lo[a,*]

[a]Institute of Plant and Microbial Biology, Academia Sinica, Taipei 115, Taiwan.

[b]Department of Life Sciences, National Central University, Taoyuan 320, Taiwan.

[c]Department of Life Sciences, National Chung Hsing University, Taichung 402, Taiwan.

[#]S-YG and L-CW contribute equally to this work

Corresponding author:

* Address correspondence to

Wan-Sheng Lo, sunnylo@gate.sinica.edu.tw;

Long-Chi Wang, lwang@dragon.nchu.edu.tw

Tel: 886-2-2787-1163, Fax: 886-2-2787-1034

Supplementary Figures

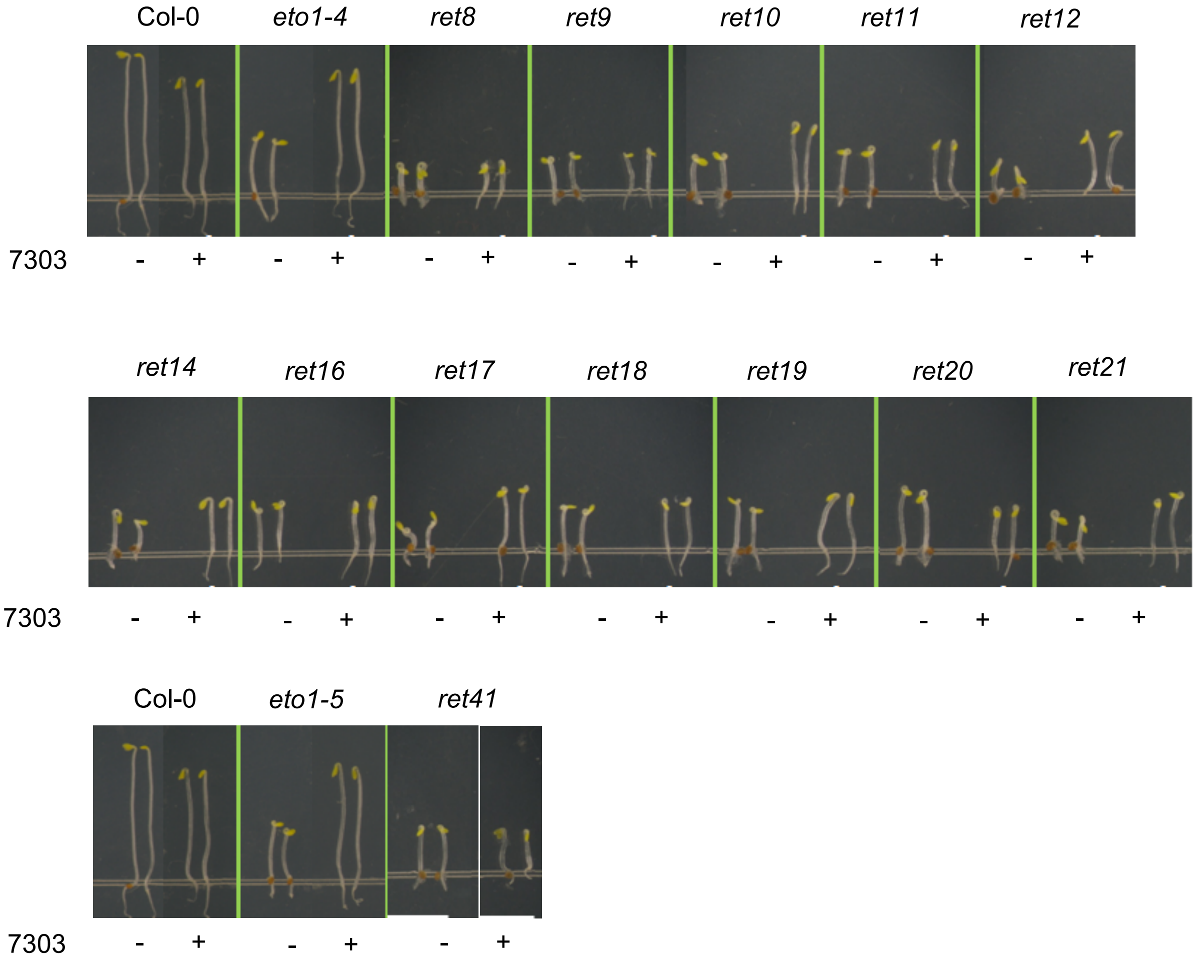

Supplementary Figure 1. Phenotypic Analysis of Arabidopsis *ret* Mutants

Phenotype of 3-day-old etiolated seedlings of Col-0, *eto1-4*, *eto1-5* and *ret* mutants grown on 1/2MS medium supplemented with (+) or without (-) 10 μM acsinone7303.

```
CTL1 (At)                           MVTIRSGSIVILVLLAVSFLALVANGE--------DKTIKVKKVRG-NKVCTQGWECSWW 51
CTL2 (At)                           MVSKPLFSLLLLTVALVVFQTGTLVNAEDSEPSSSTRKPLVKIVKG-KKLCDKGWECKGW 59
Class II chitinase (Pp)             MEKK---WLLLFSMAAATLLAVDVANGQEE----SAVKPLVKIVKG-KKVCDKGWECKGW 52
Chitinase-like protein 2 (Vv)       MEAER-SFLLIMAVAAMVVTVGGQG---------SGTKPLVKVLKGGLKVCDKGWECKGW 50
Endochitinase (Mt)                  MAKLN---AVVLLFAFFILLTTTVNGDES-----SNTKVQVKYKHG-KKYCDKGWECKGW 51
Chitinase (Br)                      MVTIRSASVFILVLLAVSTLALVANGE--------DKTIKVKKVKG-KKVCTQGWECVWW 51
Class VII chitinase precursor (Gh)  MADFKKATALILTVALLVNLALMVDADGDD-----DKKIRVRKHKG-EKQCIQGWECSYW 54
                                    *        .:: .      .                .  *:  :*  * * :****  *

CTL1 (At)                           SKYCCNQTISDYFQVYQFEQLFSKRNTPIAHAVGFWDYQSFITAAALFEPLGFGTTGGKL 111
CTL2 (At)                           SEYCCNHTISDFFETYQFENLFSKRNSPVAHAVGFWDYRSFITAAAEYQPLGFGTAGEKL 119
Class II chitinase (Pp)             SVYCCNQTISDYFQAYQFEDLFSKRNSPVAHAVGFWDYHSFITAAAEYQPHGFGTTGGKL 112
Chitinase-like protein 2 (Vv)       SKYCCNQTVSDFFQTYQFENLFAKRNTPVAHAVGFWDYRSFITAAAVYQPHGFGTAGGKL 110
Endochitinase (Mt)                  SIYCCNLTITDYFQTYQFENLFSKRNTPIAHAVGFWDYHSFINAASLFEPLGFGTTGNKT 111
Chitinase (Br)                      SEYCCNETISDYFQVYQFEQLFAKRNTPVAHAVGFWDYQSFITAAALYEPLGFGTTGGKL 111
Class VII chitinase precursor (Gh)  SKYCCNKTVSDVFQVYQFEDLFAKRNSPVAHAVGFWDYHSFILAASIYEPLGFGTTGGKR 114
                                    * **** *::* *:.****:**:***:*:*********:*** **: ::* ****:* *
                                                                     ctl1 ret9
                                                                      ▼
CTL1 (At)                           MGQKEMAAFLGHVASKTSCGYGVATGGPLAWGLCYNREMSPMQSYCDESWKFKYPCSPGA 171
CTL2 (At)                           QGMKEVAAFLGHVGSKTSCGYGVATGGPLAWGLCYNKEMSPDQLYCDDYYKLTYPCTPGV 179
Class II chitinase (Pp)             QGMKEVTAFLGHVGSKTSCGYGVATGGPTAWGLCYNKEMSPSQLYCDDYYKYTYPCSPGA 172
Chitinase-like protein 2 (Vv)       MQMKEVAAFLGHVGSKTTCGYGVATGGPLAWGLCYNKEMSPSKSYCDDDYKYTYPCTPGV 170
Endochitinase (Mt)                  TQMMEIAAFLGHVGSKTSCGYGVATGGPLAWGLCYNHEMSPAQTYCDDYYKLTYPCTPGA 171
Chitinase (Br)                      MGQKEMAAFLGHVASKTSCGYGVATGGPLAWGLCYNREMSPSQSYCDETWKFKYPCSPGA 171
Class VII chitinase precursor (Gh)  MQMKEVAAFLAHVGAKTSCGDGVIDGGPLAWGLCFKREMSPSQDYCDDYYKYMYPCAPGA 174
                                        *::***.**.:**:** **  *** *****:::**** : ***: :*  ***:**.

CTL1 (At)                           EYYGRGALPIYWNFNYGAAGEALKADLLNHPEYIEQNATLAFQAAIWRWMTPIKRAQPSA 231
CTL2 (At)                           SYHGRGALPVYWNYNYGQTGEALKVDLLSHPEYLENNATLAFQAAIWRWMTPPKKHLPSA 239
Class II chitinase (Pp)             SYHGRGALPLYWNYNYGETGDALKVDLLNHPEYIEQNATLAFQAAIWRWMTPVKKNIPSA 232
Chitinase-like protein 2 (Vv)       EYFGRGALPIYWNYNYGEAGEALKVDLLNHPEYIEQNATLAFQAAIWRWMTPVKKQQPSA 230
Endochitinase (Mt)                  EYYGRGAIPIYWNYNYGAAGEALKVNLLDHPEYIEQNATLAFQAAIWKWMTPIKKSQPSA 231
Chitinase (Br)                      EYYGRGALPIYWNFNYGAAGEALKADLLNHPEYIEQNASLAFQAAIWRWMTPIKKAQPSA 231
Class VII chitinase precursor (Gh)  QYYGRGALPIYWNYNYGAAGDGIKVDLLHHPEYLEQNATIAFQAAIWRWMTPIKKNQPSA 234
                                    .*.****:*:***:*** :*:.:*.:** ****:*:**::*******:**** *:  ***

CTL1 (At)                           HDIFVGNWKPTKNDTLSKRGPTFGSTMNVLYGEYTCGQG-SIDPMNNIISHYLYFLDLMG 290
CTL2 (At)                           HDVFVGKWKPTKNDTAAKRTPGFGATINVLYGDQICNSGFDNDEMNNIVSHYLYYLDLIG 299
Class II chitinase (Pp)             HDVFVGKWKPTKNDTLSKRVPGFGTTINVLYGDQVCGQG-DVDSMNNIVSHYLYYLDKIG 291
Chitinase-like protein 2 (Vv)       HDVFVGTWKPTKNDTLAKRIPGFGATMNVLYGDSVCGQG-DVDSMNNIVSHYQYYLDLMG 289
Endochitinase (Mt)                  HDAFVGNWKPTKNDTMENRVPGFGATMNILYGEGVCGQG-DVDSMNNIVSHYLYYLDLLG 290
Chitinase (Br)                      HDIFVGNWKPTKNDTLSKRGPTFGTTMNVLYGEYTCGQG-DIEPMNNIVSHYLYFLDLLG 290
Class VII chitinase precursor (Gh)  HDIFVGNWKPTKNDTEEKRGPTFGSTMNVLYGDYTCGQG-DIDPMNIIISHYLHYLDLLG 293
                                    ** ***.********  :* * **:*:*:***:  *..* . : ** *:*** ::** :*

CTL1 (At)                           IGREDAGPNDELSCAEQKPFNPSTVP--SSSSS- 321
CTL2 (At)                           VGREEAGPHEKLSCADQEPFSSSSSAPPSSGSSS 333
Class II chitinase (Pp)             VGREEAGPHDVLSCAEQKAFQPSSSS--SSSSS- 322
Chitinase-like protein 2 (Vv)       VGREEAGPHEVLTCAEQEAFNPSSSS--SSSSSS 321
Endochitinase (Mt)                  VGRERAGTHDVLTCAEQRPFNPNTKL---AAS-- 319
Chitinase (Br)                      IGREDAGPNEELSCAEQKAFNPSTAP--TSSSS- 321
Class VII chitinase precursor (Gh)  VGREEAGPHEELSCAEQKAFNPTPAP--PAASAS 325
                                    :*** **.:: *:**:*..*....     :.*
```

Supplementary Figure 2. Multiple sequence alignment of CTL1 homologs in selected plant species.

Protein sequences of Arabidopsis thaliana (At) *CTL1*, *CTL2* and homologous proteins from Pp (*Pyrus pyrifolia*, ACM45714), Vv (*Vitis vinifera*, XP_002276563), Mt (*Medicago truncatula*, XP_003621680), Br (*Brassica rapa*, ABV89660), Gh (*Gossypium hirsutum*, AAP80800) aligned by Clustal Omega. Arrowhead indicates the position of highly conserved Cys residue that is mutated as *ctl1*^{ret9} allele. The conserved amino acid residues are underlined. "." ":" and "*" mark residues with low, moderate and high conservation, respectively. The numbers along the right of this figure indicate amino acid positions.

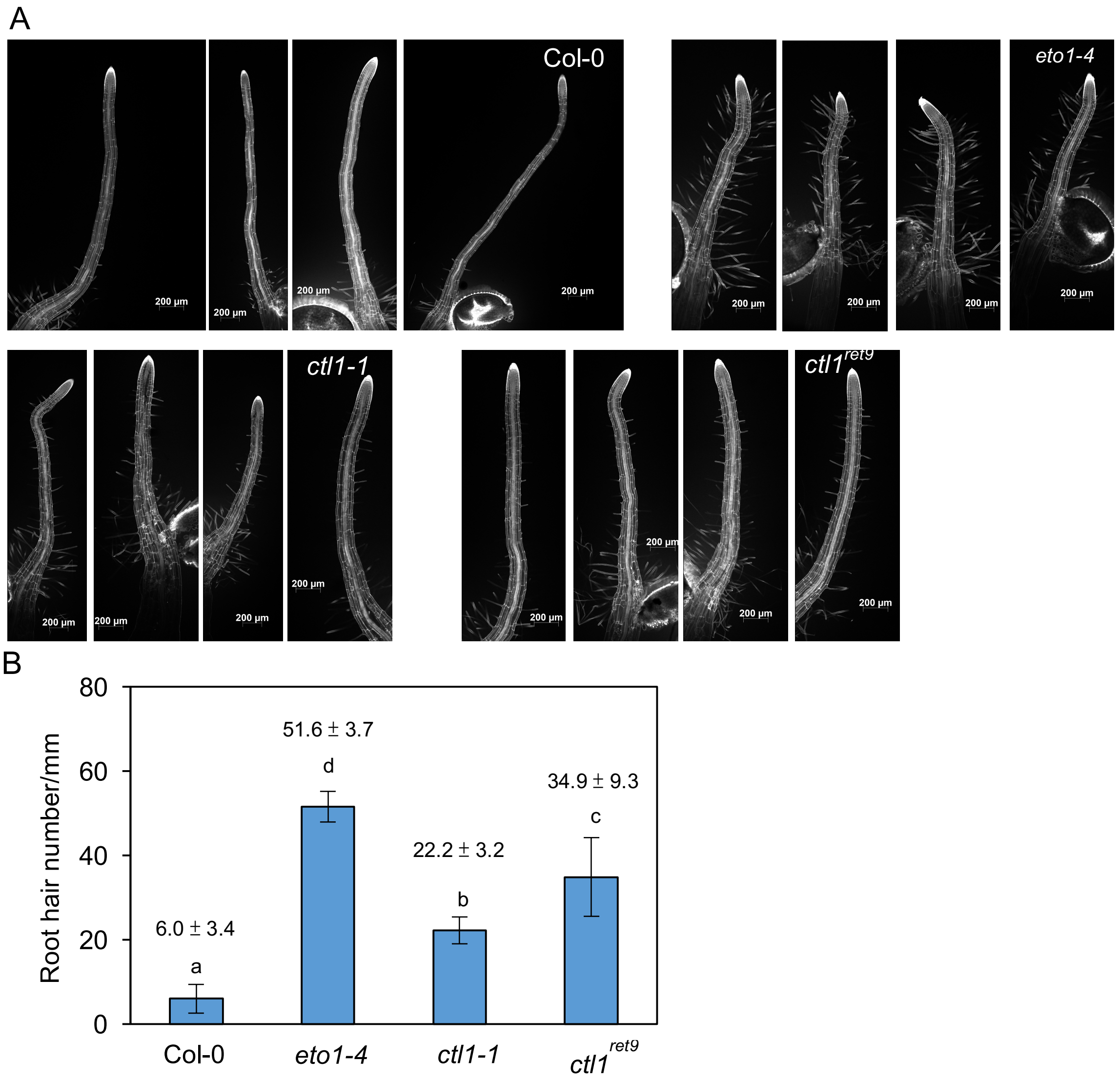

Supplementary Figure 3. The root hair density of *ctl1* mutants.

(A) Confocal microscopy of roots from 3-day-old etiolated seedlings of Col-0, *eto1-4*, *ctl1*^{ret9} and *ctl1-1* stained with propidium iodide (PI). Bar = 200 µm. (B) Root hair density (number of hairs per mm) for all genotypes in (A). Data are mean ± SD (n=4). Different lowercase letters indicate statistical significance based on ANOVA with Duncan post hoc test (p<0.05).

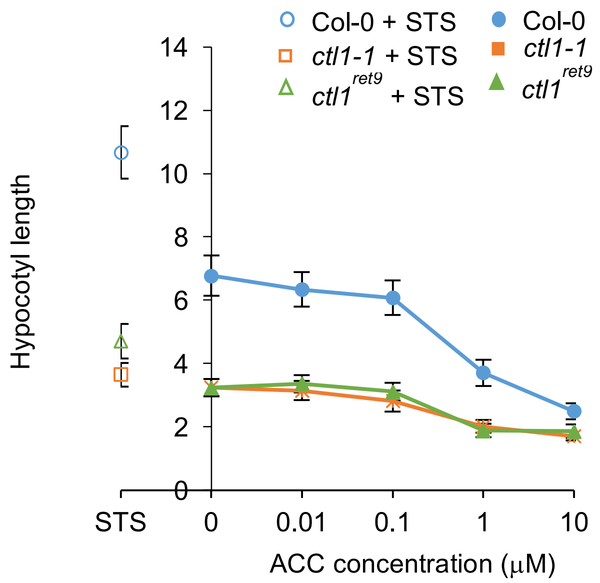

Supplementary Figure 4. The sensitivity of hypocotyl elongation to ACC in etiolated seedlings.

The hypocotyl length of 3-d-old etiolated seedlings of Col-0 (circle), *ctl1*^{ret9} (triangle) and *ctl1-1* (square) treated with 10 μM STS (open symbols, STS) or AVG (5 μM) combined with different ACC concentrations ranging from 0 to 10 μM (filled symbols). Data are mean ± SD of at least 25 measurements for each line/treatment (n≥25).

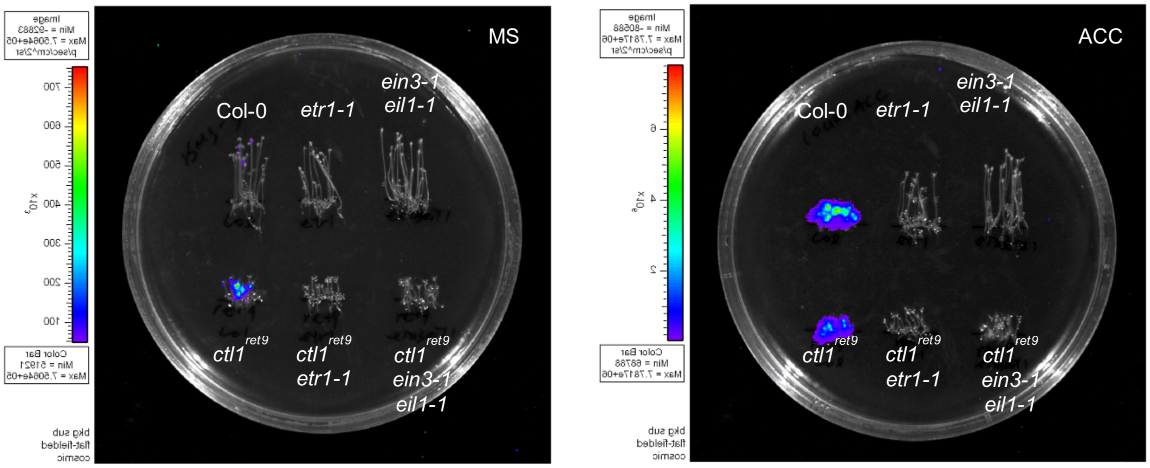

Supplementary Figure 5. The luciferase activity of 5x*EBS-LUC* reporter gene in $\mathit{ctl1}^{\mathit{ret9}}$ and ethylene-insensitive mutants.

Images of 3-day-old etiolated seedlings with luciferase activity (35Smin-*5xEBS::LUC*) in Col-0 and combination mutants of $\mathit{ctl1}^{\mathit{ret9}}$, *etr1-1* and *ein3-1 eil1-1*, germinated on 1/2MS agar medium without or with 10 μM ACC. The superimposed pseudocolor represents the photons emitted by the live cells with luciferin treatment (2 mM); the color scale bar on the right shows the photon counts (photon/s/cm^2).

Supplementary Table 1. Average hypocotyl length of Col-0 and *ret9* mutant with treatment of STS, AVG or 7303

Treatment	Average hypocotyl length (mm)				
	Col-0	*eto1-4*	*ctl1-1*	*ctl1*^{ret9}	*ret9*
MS	9.6 ± 1.4	4.0 ± 0.6	3.8 ± 0.5	2.9 ± 0.6	1.6 ± 0.3
STS	10.9 ± 1.2	10.8 ± 1.5	4.4 ± 0.7	3.8 ± 0.7	3.4 ± 0.5
AVG	9.8 ± 1.3	10.1 ± 1.1	4.7 ± 0.8	3.7 ± 0.6	3.7 ± 0.6
7303	7.7 ± 1.0	7.1 ± 0.9	2.4 ± 0.3	2.2 ± 0.4	1.8 ± 0.3

Data are mean ± SD of at least 25 3-day-old etiolated seedlings for each treatment (n≥25). The concentrate of STS, AVG and 7303 is 10, 5 and 10 μM, respectively.

Supplementary Table 2. Effect of acsinone7303 on ethylene production in *ret9* mutant

	Ethylene (nL/L/seedlings/day)				
	Col-0	*eto1-4*	*ctl1-1*	*ctl1*^{ret9}	*ret9*
MS	0.4 ± 0.2	5.9 ± 1.1	0.8 ± 0.2	0.6 ± 0.2	7.9 ± 0.9
7303	0.3 ± 0.1	0.6 ± 0.2	0.6 ± 0.3	0.6 ± 0.3	1.4 ± 0.3

3-day-old etiolated seedlings of Col-0 and mutants were grown in capped vials filled MS agar medium with or without acsinone7303μM. Data are mean ± SD of 3 biological replicates with 35 seedlings.

Supplementary Table 3. Primers used in the study.

Primer	Sequence (5'→3')	Used for
pCambia1300-g*CTL1*_F	GTTGGGATCCGTTACTGTTGGAAAACAAGGG (*BamH*I)	Complementation
pCambia1300-g*CTL1*_R	CTTCGTCGACCGTGCCTGATGTTTACAGCG (*Sal*I)	Complementation
dCAPS_*ret9*_F	GTTGCAACAGGAGGGCCTTTAGCTTGGGGTCAGT (*Sac*I)	$ctl1^{ret9}$ genotyping
dCAPS_*ret9*_R	CCATCTCCAGATTGCAGCTT	$ctl1^{ret9}$ genotyping
dCAPS_*eto1-4*_F	CAGGCTGATTGCTGGATGCAGCTGTATGATCCTTG (*Sty*I)	*eto1-4* genotyping
dCAPS_*eto1-4*_R	CGCTTCAAACGACCTTTGGATGG	*eto1-4* genotyping
dCAPS_*etr1-1*_F	CTGTCTACGCTACGTTCTCG	*etr1-1* genotyping
dCAPS_*etr1-1*_R	GTCCATAAGTTAATAAGATGAGTTGATCGA (*Cla*I)	*etr1-1* genotyping
*ein2-47*_F	AAG TCA AGG ACA CGG TGA ATG	*ein2-47* genotyping
*ein2-47*_R	GGG TGC ATA TGG TAA CAC CAC	*ein2-47* genotyping
CAPS_*ein3-1*_F	GCTCAAGGCTTTGTTTATGGG	*ein3-1* genotyping
CAPS_*ein3-1*_R	CTGCAAACATTTAGATTGCCTC (*Bst*I)	*ein3-1* genotyping
*eil1-1*_F	CTGAGAAGGGTAAGCCTGTG	*eil11-1* genotyping
*eil1-1*_R	CTTCAACGTCATACTCGCTAC	*eil11-1* genotyping
*5xEBS::LUC*_F	TCACCGCTTCCCTCATGATG	*5xEBS:LUC*
*5xEBS::LUC*_R	CACGGTAGGTGCGAAATGC	*5xEBS:LUC*
ACO1 (AT2G19590)_F	TTGCTACGTTTTACAATCCGGC	Quantitative RT-PCR
ACO1 (AT2G19590)_R	AGGTAGTCTTGAAAACGGTAGCCA	Quantitative RT-PCR
ACO2 (AT1G62380)_F	TCTACGTTCGTCACCTCCCTCA	Quantitative RT-PCR
ACO2 (AT1G62380)_R	CTCTTACCAAAGTCTTTCATGGCC	Quantitative RT-PCR
ERF1_F	ATTCTTTCTCATCCTCTTCTTCT	Quantitative RT-PCR
ERF1_R	CGAATCTCTTATCTCCGCCG	Quantitative RT-PCR

EDF1_F	TCACAAACACAACAAATATGGAATACAG	Quantitative RT-PCR
EDF1_F	GCTTTGGAGTAGAGATGGAGAG	Quantitative RT-PCR
*UBQ10 (AT4G05320)*_F	TTCCTTGATGATGCTTGCTC	Quantitative RT-PCR
*UBQ10 (AT4G05320)*_R	TTGACAGCTCTTGGGTGAAG	Quantitative RT-PCR
